# Subclinical Hypothyroidism in PCOS: Impact on Presentation, Insulin Resistance, and Cardiovascular Risk

**DOI:** 10.1155/2016/2067087

**Published:** 2016-07-12

**Authors:** Qun Yu, Jin-Bei Wang

**Affiliations:** Department of Obstetrics, Yantai Yuhuangding Hospital, Yantai, Shandong 264000, China

## Abstract

*Aim of Study*. To assess status of thyroid function and thyroid disorders particularly subclinical hypothyroidism (SCH) in subjects with polycystic ovarian syndrome (PCOS) and impact of SCH on various clinical and biochemical parameters and cardiovascular risk in PCOS.* Methods*. Hundred females diagnosed with PCOS as per Rotterdam criteria and 100 normal controls were recruited and were subjected to elaborate anthropometric, clinical, and biochemical assessment.* Results*. Notable findings included significantly higher frequency of subjects with subclinical hypothyroidism (*p* = 0.0002), autoimmune thyroiditis (*p* < 0.001), and goitre (*p* = 0.02) in polycystic ovarian syndrome subjects compared to control subjects. Further SCH PCOS subjects were found to harbor significantly higher HOMA-IR (*p* < 0.05) and frequency of subjects with dyslipidemia (*p* < 0.05) compared to both euthyroid PCOS and euthyroid control subjects. Though frequency of subjects with cardiovascular risk factors was higher in SCH PCOS group than euthyroid PCOS group, it failed to reach statistical significance.* Conclusion*. We concluded that PCOS is associated with high incidence of SCH and AIT compared to normal population and SCH poses increased risk of cardiovascular disorder in PCOS.

## 1. Introduction

Polycystic ovarian syndrome (PCOS) is characterized by various menstrual and hormonal irregularities culminating in anovulation, infertility, and hyperandrogenism [1, 2]. Insulin resistance (IR) and hyperandrogenism are amongst the most common endocrine irregularities encountered in PCOS. More than half of subjects with PCOS are associated with IR, hyperglycemia, weight gain, and finally metabolic syndrome (MBS) [1, 3]. A similar picture is also shared by hypothyroidism due to associated hyperglycemia, raised levels of sex hormone binding globulin (SHBG), and dyslipidemia [2, 4, 5]. Notably, cystic changes with raised ovarian mass have also been reported in hypothyroidism. Two facts make the picture more interesting, first that both have different etiopathology and second that reportedly thyroid disorders are more common in PCOS subjects [6, 7]. Hypothyroidism by virtue of raised thyrotropin releasing hormone (TRH) causes altered follicle stimulating hormone (FSH)/luteinizing hormone (LH) ratio and raised dehydroepiandrosterone (DHEA-S) levels. Also, excess thyroid stimulating hormone (TSH) causes stimulation of FSH receptor. The coexistence of hypothyroidism and PCOS has been related to complex pathophysiological changes caused by obesity and IR observed in the PCOS, though not conclusively [8]. On this background the aim of our study was to assess a status of thyroid function and thyroid disorders particularly subclinical hypothyroidism in subjects with polycystic ovarian syndrome. We also tried to assess the impact of subclinical hypothyroidism on various clinical and biochemical parameters including those indicating insulin resistance and cardiovascular risk in PCOS.

## 2. Materials and Methods

Present study was a hospital based case-control study, cases being patients attending outpatient clinics of Obstetrics and Gynecology and infertility clinic of Yantai Yuhuangding Hospital in age group 13–45 years, with complaints of hirsutism and/or oligomenorrhea or infertility and diagnosed as PCOS. The study was explained to all such subjects in detail and first 100 subjects providing consent to enter the study were included in the study. In case of minor subjects, consent was obtained from parents/guardian. Hundred age- and sex-matched healthy subjects amongst relatives of the patients or hospital staffs were included as control after informed written consent. The study was approved by the institutional ethical committee and was performed in accordance with the “Declaration of Helsinki.”

PCOS was defined using Rotterdam criteria [9] which included any 2 of following 3 to be present: (1) abnormal menstruation including amenorrhea (absence of menstrual cycles in the last 6 months) or oligomenorrhea (cycles >35 days); (2) hyperandrogenism either clinical (hirsutism defined by Ferriman and Gallwey score > 7 by physician [10] and/or acne and/or alopecia (androgenic pattern) [11]) or biochemical (testosterone > 2.0 nmol/L); (3) presence of polycystic ovaries (follicles 2–9 mm in diameter and ≥12 in number or ovarian volume ≥ 10 cm^3^) on transabdominal pelvic ultrasonography (USG), after ruling out other differential diagnoses as congenital adrenal hyperplasia (CAH), virilizing tumors, Cushing's syndrome, and prolactinomas [12]. Adrenocorticotropin-stimulated 17-hydroxyprogesterone test and dexamethasone suppression test and/or 24-hour urinary cortisol excretion, if hypercortisolism is clinically suspected, were used to exclude other etiologies for hyperandrogenism. Subjects with known history of endocrine dysfunctions like Cushing's syndrome, hyperprolactinemia, and gonadal or adrenal neoplasm were excluded. 100 age-matched healthy females without the history of PCOS and known thyroid abnormality were selected as controls.

A detailed history was elucidated and all participants were subjected to elaborate anthropometric and clinical examination. 5 mL of venous blood was obtained and was used for estimation of fasting plasma glucose, lipid profile, and hormonal analysis. Blood sample was taken on day 2 or 3 of the menstrual cycle (in women with regular menstrual cycles) or on day 2 or 3 of the induced withdrawal bleeding (in amenorrheic women). Withdrawal bleeding was induced in amenorrheic women with oral progestin (medroxyprogesterone acetate, 5 mg tablets) twice daily for 5 days. Pregnancy was excluded by a negative serum pregnancy test in all subjects. Hormonal analysis included serum free triiodothyronine (T3), free tetraiodothyronine (T4), TSH, anti-thyroperoxidase antibody (anti-TPO ab), LH, FSH, prolactin, insulin, free testosterone, progesterone, estradiol, and SHBG-S using Cobas e-411 analyzer (Roche Diagnostics Ltd., Mannheim, Germany) by electrochemiluminescence immunoassay (ECLIA) method as per manufacturer's protocol (intra-assay and interassay CV <5.0%). Lipid profile and plasma glucose were estimated on Cobas c-311 clinical chemistry analyzer (Roche Diagnostics Ltd., Mannheim, Germany) by standard methods as per manufacturer's protocol.

Dyslipidemia was considered when levels of LDL cholesterol >130 mg/dL or triglyceride (TG) >150 mg/dL or HDL <40 mg/dL were found. Subjects were considered hypertensive if systolic blood pressure was found to be >140 mmHg or diastolic blood pressure was found to be >90 mmHg.

USG thyroid was performed, using 7.5 MHz transducer with Duplex sonography, using Quadroline 505 (General Electric, Frankfurt, Germany). If echogenicity of thyroid was found to be equal or lower than surrounding tissue, it was labeled to be hypoechoic. The following formula was used to calculate HOMA-IR: fasting insulin (*μ*U/L) × fasting glucose (mg/dL)/405.

Autoimmune thyroiditis (AIT) was diagnosed based on the presence of anti-TPO ab along with hypoechoic thyroid on radiological examination ultrasonography (USG). Subclinical hypothyroidism (SCH) was diagnosed when TSH levels were >4.25 mIU/mL and T3 and T4 levels were found within the normal range. Hypothyroidism and hyperthyroidism was diagnosed based on recommendations by American Thyroid Association.

*Statistical Analysis*. Data was expressed as mean ± SD and percentage. Kolmogorov-Smirnov analysis was done to assess the linearity of data. Student's t-test was used to access statistical significance between two groups. To assess the significance of difference between more than two groups, ANOVA followed by post hoc analysis using Tukey's HSD was used when parameters were found to be normally distributed and for parameters with nonnormal distribution Kruskal-Wallis test followed by post hoc analysis with Bonferroni's test was used. Chi-square analysis and Fischer's exact test were used to study frequency distribution in different categories. *p* value < 0.05 was considered to be statistically significant. SPSS Version 12 and Microsoft Excel (2007) were used to perform statistical calculations. Sample size for study satisfactorily exceeded the calculated sample size with expected power of study to be 90% and type 1 error to be 5%.

## 3. Results

Total of 100 subjects diagnosed with PCOS and 100 controls were assessed in current study.  Table 1 denotes characteristics of study groups. PCOS and control groups were matched for age and BMI. Frequency of hirsutism and Ferriman and Gallwey score were found to be significantly higher in PCOS subjects compared to controls (*p* < 0.001). As expected it was found that PCOS subjects were having significantly higher LH (*p* < 0.001), LH/FSH ration (*p* < 0.01), and HOMA-IR (*p* < 0.001) compared to controls. On evaluation of thyroid profile significantly higher TSH (*p* < 0.001) and anti-TPO ab (*p* < 0.01) levels and significantly lower T3 (*p* < 0.001) levels compared to controls were found in PCOS subjects. Though T4 levels were found to be slightly lower in PCOS (1.01 ± 2.8 ng/dL) compared to controls (1.21 ± 2.9 ng/dL) the difference failed to reach statistical significance (*p* = NS). Also, frequency of hypoechoic thyroid on USG was found to be more amongst PCOS subjects (34%) compared to controls (7%) (*p* < 0.01). Lipid parameters (total cholesterol, TG, and LDL) and IR were found to be significantly higher, while HDL was found to be significantly lower in PCOS subjects compared to control subjects.

Further, it was noted that the frequency of thyroid disorders was significantly higher amongst PCOS subjects. While PCOS group was harboring 3% overt hypothyroid, 25% autoimmune thyroiditis (AIT) subjects, 27% subjects with subclinical hypothyroidism (SCH), and 25% subjects with goitre, control group was found to have only 2% AIT (*p* < 0.001), 8% SCH (*p* = 0.0002), and 2% goitre (*p* < 0.001). While 68% of PCOS subjects were euthyroid, 91% amongst control subjects were euthyroid (*p* < 0.0001) (Table 2).

Clinical and biochemical parameters were also compared amongst euthyroid PCOS (*n* = 68), SCH PCOS (*n* = 27), and euthyroid controls (*n* = 91) (Table 3). Mean age of subjects with subclinical hypothyroidism was found to be significantly higher compared to euthyroid PCOS subjects (*p* = 0.01). No significant difference was noted in BMI, LH, FSH, LH/FSH ratio, free T3, free T4, estrogen, and progesterone but, notably, many clinical (BMI, hypertension, hirsutism, and Ferriman and Gallwey score) and biochemical (TSH, free testosterone, progesterone, HOMA-IR, and lipid profile) parameters were significantly different between three groups. Amongst other notable findings anti-TPO ab, HOMA-IR, and lipid profile (total cholesterol, LDL, and TG) were significantly higher in SCH PCOS compared to euthyroid PCOS subjects.

To assess the impact of hypothyroidism in PCOS on cardiovascular risk we studied the distribution of cardiovascular risk factors in SCH PCOS, euthyroid PCOS, and euthyroid control groups (Table 4). We found that while frequency of all the risk factors was significantly different amongst three groups (*p* = 0.008 for hypertension, *p* < 0.0001 for HOMA-IR, and *p* < 0.0001 for dyslipidemia), HOMA-IR and dyslipidemia were found to be significantly higher in SCH PCOS compared to subclinical hypothyroidism.

## 4. Discussion

We examined 100 PCOS and 100 control subjects for various clinical, biochemical, and radiological parameters (Table 1) and found a significantly higher frequency of thyroid related disorders in PCOS subjects compared to control subjects. Hypothyroidism was the most common thyroid dysfunction with frequency of SCH being 27% and that of overt hypothyroidism 3%. A few studies have previously analyzed SCH in PCOS subjects. In a study done by Enzevaei et al. in Iran, they have observed 25.5% of subjects having SCH [13], while, in a study conducted by Sinha et al. in Indian population, 22.5% subjects with PCOS were detected to be having subclinical hypothyroidism [7]. Reports from remote past have also indicated elevated TSH levels, both basal and TRH-induced [14].

Significantly higher (*p* < 0.001) frequency of AIT in PCOS subjects (25%) compared to controls (2%) reported in our study is in accordance with previous literature. Frequency of AIT was found to be 20.6% (overt AIT) and 26.9% (thyroid specific antibody positive) in one of the first prospective multicenter studies on thyroid function in PCOS conducted by Janssen et al. in German population [15]. Kachuei et al. have also found significantly higher (*p* = 0.04) prevalence of anti-thyroglobulin antibody (anti-Tg ab) in PCOS subjects compared to normal controls in Iranian population [16]. But in a study including nonorgan specific antibodies in PCOS subjects by Petrikova et al. prevalence of anti-TPO ab but not AIT was found to be significantly higher in PCOS subjects compared to controls [17]. Probable mechanism of subclinical hypothyroidism in PCOS has been suggested to course through associated obesity and high BMI. Associated proinflammatory condition and IR, through yet undefined mechanism, may be leading to decreased deiodinase-2 activity, thus leading to relative low T3 and higher TSH levels [18]. Alternate mechanism indicating obesity leading to increased leptin levels which stimulate hypothalamus causing increased TRH secretion has also been proposed [19]. Any of these or both these pathways working simultaneously may be an explanation to the high incidence of SCH in PCOS. High incidence of AIT can probably be attributed to high estrogen and low progesterone present in PCOS, similarly as encountered in menopause [20]. High estrogen leads to increased *ϒ*-interferon expression TH-1 cells and increased expression of IL-6, which is a potent mediator of autoimmunity, in T-cell. These inflammatory mediators are further proposed to induce expression of many functional FAS molecules in thyroid follicles. Further followed by thyroid destruction via apoptosis, either by thyroid cell FAS ligand (Fasl) or by Fasl armed TH-1 cells and T-cell proliferation [21, 22].

Impact of SCH on clinical and biochemical characteristics of study population was also assessed (Table 3). One of the notable findings was that though SCH PCOS group and euthyroid PCOS group were matched for BMI, HOMA-IR was found to be significantly higher in SCH PCOS group. Also, HOMA-IR in euthyroid PCOS was significantly higher compared to euthyroid controls. Enzevaei et al. have reported findings contradictory to ours stating that SCH in PCOS does not have a significant impact on IR (*p* = 0.74), but they have considered HOMA-IR >3.2 to be cut off for insulin resistance [13]. Also, Ganie et al. in a study in Indian population reported no significant difference in IR between SCH and euthyroid subjects with PCOS [23]. Celik et al. in their study reported that in Turkey population hypothyroid and euthyroid group in PCOS were not significantly different in terms of IR after removing confounding impact of BMI [24]. Interestingly all these studies reported higher HOMA-IR in SCH PCOS subjects compared to euthyroid PCOS and also cut-off for IR varied amongst these studies. But Mueller et al. in their study reported association between raised TSH levels and IR independent of BMI [2]. Also in some studies, HOMA-IR has been found to be increased in SCH group in subjects without PCOS [25, 26]. Also, Abd El-Hafez et al. reported a significant correlation between TSH levels and insulin resistance in PCOS subjects [27].

Increased insulin resistance in setting of SCH has been attributed previously to impaired translocation of GLUT-4 insulin receptors present in skeletal muscle and adipose tissue [28].

We have also found significantly altered lipid profiles and higher frequency of dyslipidemia in SCH PCOS group compared to euthyroid PCOS group. Altered lipid profile has been rampantly noted amongst PCOS subjects and is supposed to be due to IR [29, 30]. Other environmental and genetic factors are also supposed to play an important role in the severity of both IR and dyslipidemia [31]. One of these could be associated subclinical hypothyroidism owing to already discussed mechanisms. The overall picture finally gives rise to MBS in some subjects which is itself another risk factor for cardiovascular ailments [32]. We have in our study detected a significant higher frequency of cardiovascular risk factors in PCOS subjects compared to controls. The frequency of these risk factors was found to be significantly higher in SCH PCOS group compared to euthyroid PCOS group; also both PCOS groups showed significantly higher frequency of both the risk factors compared to euthyroid controls (Table 4). In similar studies Tuzcu et al. [26] and Al Sayed et al. have reported significantly higher total cholesterol and LDL in SCH subjects compared to controls, but the population was not limited to PCOS subjects [26, 33]. Contradictory to our findings, Enzevaei et al. found no significant difference in lipid profiles in SCH and euthyroid PCOS group [13]. Laway et al. too failed to get any significant difference in lipid profile in SCH and euthyroid PCOS subjects [34]; they both have not compared the frequency of risk factors, neither was euthyroid control population included in comparison.

The notable differences in findings regarding IR and lipid profile in our study from various previous authors can also be attributed to different populations included in the study.

It should be noted that, unlike most previous studies in past which have included controls from subjects visiting the hospital for conditions other than PCOS, we have specifically included normal healthy controls which better represent the population. Further, results of the studies should be interpreted taking into notice that we have performed only anti-TPO ab and not anti-Tg ab, but we have also used USG thyroid as an additional investigation to support our diagnosis of AIT. Results should also be interpreted considering the fact that relatively less number of subjects [25] were left in PCOS SCH group and further studies with higher sample size are warranted.

## 5. Conclusion

Based on findings of our study we conclude that PCOS is associated with high incidence of thyroid disorders compared to normal population specifically SCH and AIT. Cardiovascular risk factors, namely, hypertension, dyslipidemia, and IR, are significantly higher in PCOS subjects compared to normal control subjects. These risk factors barring hypertension were further found to be significantly higher in PCOS subjects with SCH compared with euthyroid PCOS subjects. Further it was also noted that SCH status of PCOS subject does not cause significant changes in other biochemical parameters with notable exception of lipid profile.

## Figures and Tables

**Table 1:** Characteristics of study population.

Variable	PCOS (*n* = 100) (mean ± SD)	Control (*n* = 100) (mean ± SD)	*p* value
Age (years)	27.4 ± 5.4	23.3 ± 4.1	NS
BMI (Kg/m^2^)	31.2 ± 8.3	29.2 ± 5.1	NS
Hypertension (%)	18 (18)	5 (5)	<0.001
Hirsutism (%)	76 (*n* = 76)	13 (*n* = 13)	<0.001^*∗*^
Ferriman and Gallwey score	19.31 ± 9.7	8.32 ± 4.8	<0.001^*∗*^
LH (mIU/mL)	12.72 ± 4.9	10.19 ± 4.1	<0.001^*∗*^
FSH (mIU/mL)	5.01 ± 2.1	4.26 ± 2.1	0.01
LH/FSH ratio	2.7 ± 0.8	1.05 ± 1.02	<0.01^*∗*^
Free T3 (pg/mL)	2.61 ± 1.4	3.48 ± 0.8	0.03
Free T4 (ng/dL)	1.17 ± 1.2	1.21 ± 2.9	NS
TSH (mIU/mL)	5.11 ± 22.7	2.9 ± 3.2	<0.001^*∗*^
Anti-TPO ab (IU/mL)	76.23 ± 23.4	20.14 ± 12.4	<0.001^*∗*^
Free testosterone (pg/mL)	21.13 ± 9.2	12.4 ± 6.1	<0.001^*∗*^
Estradiol (pg/mL)	62.21 ± 31.6	67.34 ± 45.21	NS
Progesterone (ng/mL)	2.4 ± 1.8	9.1 ± 5.2	<0.001^*∗*^
HOMA-IR	3.6 ± 1.7	1.7 ± 1.0	<0.001^*∗*^
Hypoechoic USG (%)	34 (34)	7 (7)	<0.01^*∗*^
Total cholesterol (mg/dL)	232 ± 31.4	172 ± 26.7	0.02^*∗*^
Triglycerides (mg/dL)	124 ± 21.3	86.9 ± 12.4	0.01^*∗*^
LDL Chol. (mg/dL)	143 ± 19.7	112 ± 26.1	0.01^*∗*^
HDL Chol. (mg/dL)	39.4 ± 17.3	56.9 ± 6.7	0.01^*∗*^

^*∗*^ *p* < 0.05; statistically significant.

**Table 2:** Frequency of thyroid disorders in study groups.

Thyroid disorder	PCOS *n* (%)	Control *n* (%)	*p* value
Overt hypothyroid	3 (3)	0	0.01^*∗*^
Autoimmune thyroiditis	25 (25)	2 (2)	<0.001^*∗*^
Subclinical hypothyroidism	27 (27)	8 (8)	0.0002^*∗*^
Goitre	25 (25)	2 (2)	0.02^*∗*^
Euthyroid	68 (68)	91 (91)	<0.0001^*∗*^
Hyperthyroid	2 (2)	1 (1)	NS

^*∗*^ *p* < 0.05; statistically significant.

**Table 3:** Clinical and biochemical characteristics of SCH PCOS, euthyroid PCOS, and euthyroid control subjects.

	SCH PCOS (*n* = 27) (mean ± SD)	Euthyroid PCOS (*n* = 68) (mean ± SD)	Euthyroid control (*n* = 91) (mean ± SD)	*p* value
Age (years)	29.21 ± 5.9	26.37 ± 4.5	28.1 ± 3.9	NS^b^
BMI (Kg/m^2^)	32.70 ± 4.9	30.91 ± 5.1	27.3 ± 4.7	0.01^a^
Hypertension *n* (%)	7 (25.9)	11 (16.17)	5 (5.5)	0.008^a^
Hirsutism (%)	25 (92%)	51 (75%)	4 (5%)	0^a^
Ferriman and Gallwey score	20.91 ± 5.3	18.37 ± 4.9	8.21 ± 4.3	0.1^a^
LH	13.12 ± 7.6	11.39 ± 6.8	5.81 ± 3.7	0.02^a^
FSH	4.7 ± 1.9	4.8 ± 1.8	4.41 ± 1.9	NS^b^
LH/FSH ratio	2.9 ± 0.5	2.7 ± 0.7	1.01 ± 0.29	NS^b^
Free T3 (pg/mL)	2.42 ± 1.3	2.82 ± 1.3	3.73 ± 0.7	NS^b^
Free T4 (ng/dL)	1.02 ± 1.0	1.23 ± 1.1	1.32 ± 1.6	NS^b^
TSH (mIU/mL)	7.2 ± 3.5	3.1 ± 1.5	3.2 ± 1.2	<0.01^a^
Anti-TPO ab (IU/mL)	139.54 ± 51.7	32.59 ± 5.1	19.34 ± 10.3	<0.001^c^
Free testosterone (pg/mL)	23.09 ± 6.3	18.34 ± 7.9	12.2 ± 5.7	<0.01^a^
Estradiol (pg/mL)	59.31 ± 18.6	63.83 ± 26.7	74.24 ± 29.26	NS^b^
Progesterone (ng/mL)	2.5 ± 0.6	2.9 ± 1.2	7.4 ± 4.3	NS^b^
HOMA-IR	4.2 ± 1.1	2.8 ± 1.4	1.8 ± 0.7	0.01^d^
Total cholesterol (mg/dL)	230 ± 29.6	198 ± 23.1	159 ± 24.8	0.01^d^
Triglycerides (mg/dL)	136 ± 21.3	120 ± 16.7	84.3 ± 10.6	0.001^d^
LDL Chol. (mg/dL)	151 ± 17.3	139 ± 12.6	109 ± 20.4	0.02^d^
HDL Chol. (mg/dL)	37.4 ± 15.4	45.9 ± 9.6	60.3 ± 5.2	0.04^a^

^a^ *p* = NS between SCH PCOS and euthyroid PCOS, *p* < 0.05 between SCH PCOS and euthyroid controls, and *p* < 0.05 between euthyroid PCOS and euthyroid control group on post hoc analysis.

^b^ *p* = NS between SCH PCOS and euthyroid PCOS group, *p* = NS between SCH PCOS and euthyroid controls, and *p* = NS between euthyroid PCOS and euthyroid control group on post hoc analysis.

^c^ *p* < 0.05 between SCH PCOS and euthyroid PCOS group, *p* < 0.05 between SCH PCOS and euthyroid controls, and *p* = NS between euthyroid PCOS and euthyroid control group on post hoc analysis.

^d^ *p* < 0.05 between SCH PCOS and euthyroid PCOS group, *p* < 0.05 between SCH PCOS and euthyroid controls, and *p* < 0.05 between euthyroid PCOS and euthyroid control group on post hoc analysis.

**Table 4:** Frequency of subjects with cardiovascular risk factors in SCH PCOS, euthyroid PCOS, and euthyroid control subjects.

	SCH PCOS (*n* = 27)	Euthyroid PCOS (*n* = 68)	Euthyroid control (*n* = 91)	*p* value
Hypertension *n* (%)	7 (25.9)	11 (16.17)	5 (5.5)	0.008^a^
Insulin resistance (HOMA-IR >2.0)	27 (100)	38 (56)	15 (16.48)	<0.0001^b^
Dyslipidemia	24 (89)	31 (45.5)	13 (14.2)	<0.0001^b^

^a^ *p* = NS between SCH PCOS and euthyroid PCOS, *p* < 0.05 between SCH PCOS and euthyroid controls, and *p* < 0.05 between euthyroid PCOS and euthyroid control group.

^b^ *p* < 0.05 between SCH PCOS and euthyroid PCOS group, *p* < 0.05 between SCH PCOS and euthyroid controls, and *p* < 0.05 between euthyroid PCOS and euthyroid control group on post hoc analysis.
